# Elucidating the Efficacy of Vaccination against Vibriosis in *Lates calcarifer* Using Two Recombinant Protein Vaccines Containing the Outer Membrane Protein K (r-OmpK) of *Vibrio alginolyticus* and the DNA Chaperone J (r-DnaJ) of *Vibrio harveyi*

**DOI:** 10.3390/vaccines8040660

**Published:** 2020-11-06

**Authors:** Santha Silvaraj, Ina Salwany Md Yasin, Murni Marlina A. Karim, Mohd Zamri Saad

**Affiliations:** 1 Laboratory of Aquatic Animal Health and Therapeutics, Institute of Bioscience, Universiti Putra Malaysia, UPM, Serdang 43400, Malaysia; santha_sr@yahoo.com (S.S.); murnimarlina@upm.edu.my (M.M.A.K.); mzamri@upm.edu.my (M.Z.S.); 2 Department of Aquaculture, Faculty of Agriculture, Universiti Putra Malaysia, UPM, Serdang 43400, Malaysia; 3 Department of Veterinary Laboratory Diagnosis, Faculty of Veterinary Medicine, Universiti Putra Malaysia, UPM, Serdang 43400, Malaysia

**Keywords:** recombinant vaccine, cross-protection, immune response, juvenile seabass

## Abstract

Recombinant cell vaccines expressing the *OmpK* and *DnaJ* of *Vibrio* were developed and subsequently, a vaccination efficacy trial was carried out on juvenile seabass (~5 cm; ~20 g). The fish were divided into 5 groups of 50 fish per group, kept in triplicate. Groups 1 and 2 were injected with 10^7^ CFU/mL of the inactivated recombinant cells vaccines, the pET-32/LIC-*OmpK* and pET-32/LIC-*DnaJ*, respectively. Group 3 was similarly injected with 10^7^ CFU/mL of inactivated *E. coli* BL21 (DE3), Group 4 with 10^7^ CFU/mL of formalin killed whole cells *V. harveyi*, and Group 5 with PBS solution. Serum, mucus, and gut lavage were used to determine the antibody levels before all fish were challenged with *V. harveyi*, *V. alginolyticus*, and *V. parahemolyticus*, respectively on day 15 post-vaccination. There was significant increase in the serum and gut lavage antibody titers in the juvenile seabass vaccinated with *r*-*OmpK* vaccine. In addition, there was an up-regulation for TLR2, MyD88, and MHCI genes in the kidney and intestinal tissues of *r-OmpK* vaccinated fish. At the same time, *r-OmpK* triggered higher expression level of interleukin IL-10, IL-8, IL-1ß in the spleen, intestine, and kidney compared to *r-DnaJ*. Overall, *r-OmpK* and *r-DnaJ* triggered protection by curbing inflammation and strengthening the adaptive immune response. Vaccinated fish also demonstrated strong cross protection against heterologous of *Vibrio* isolates, the *V. harveyi*, *V. alginolyticus*, and *V. parahaemolyticus*. The fish vaccinated with *r-OmpK* protein were completely protected with a relative per cent of survival (RPS) of 90 percent against *V. harveyi* and 100 percent against *V. alginolyticus* and *V. parahaemolyticus*. A semi-quantitative PCR detection of *Vibrio* spp. from the seawater containing the seabass also revealed that vaccination resulted in reduction of pathogen shedding. In conclusion, our results suggest *r-OmpK* as a candidate vaccine molecule against multiple *Vibrio* strain to prevent vibriosis in marine fish.

## 1. Introduction

Aquaculture accounts for almost half of the world’s food fish and is the fastest growing sector of agriculture in the world [1]. The worldwide aquaculture production is reported to have increased from 28 million tons in 1998 to 50 million tons in 2007 [2]. Asian seabass (*Lates calcarifer*), also known as barramundi, is a famous aquaculture species in Southeast Asia and Australia. However, disease outbreak is a major constraint to the development of Asian seabass aquaculture. One of the major diseases affecting Asian seabass and other cultured marine teleost is vibriosis, resulting in either chronic low-grade mortalities or acute outbreaks, leading to mass mortalities. Currently, it is a region-wide problem in Asian seabass culture throughout Southeast Asia. To control vibriosis, the infected fishes were fed with antibiotic-laden feed [3] or antibiotics treatment via oral route to groups of fish sharing the same tanks or cages [4]. However, the use of antibiotics is currently discouraged due to the development of resistance to antimicrobial compounds in pathogens. Another problem created by unrestricted antibiotics use is the presence of residual antibiotics in commercialized aquaculture products, which has led to allergy and toxicity in humans [5].

Vaccination is one of the most effective and alternative methods in combating threatening diseases in fish. This preventive method can significantly reduce specific disease-related loses, resulting in a reduction of antibiotics use and is becoming an increasingly important part of aquaculture. Several types of vaccines, including DNA-based, live attenuated, and inactivated vaccines have been developed against vibriosis, and vaccines that are based on inactivated bacterial pathogens are proven to be quite efficacious in fish [6]. This explains why most bacterial vaccines used in aquaculture are inactivated vaccines [7]. Although vaccines are expected to induce long-lived, antibody-mediated immune-protection, little is known about the basic protective mechanisms elicited by the immunization of fish. The efficiency of a vaccine should not be evaluated in terms of protection alone but in terms of its ability to elicit humoral and cellular immune responses to the targeted antigen [8].

The outer membrane proteins (OMPs) of Gram-negative bacteria have important roles in the interactions between bacteria and host. Mao et al. [9] stated that a recombinant OMP from *V. harveyi* and *V. parahaemolyticus* was able to protect yellow croaker, *Pseudosciaena crocea* from infection by virulent strains of both bacteria. Moreover, DNA vaccines that use the OMP genes against *V. harveyi* have been tested for protective ability in Asian seabass. A vaccination trial conducted by Li et al. [10] using an inactivated vaccine in *Sparus sarba* against *V. alginolyticus* revealed that the circulating lymphocyte counts were significantly elevated in the fish immunized with the bacterins.

In this study, we used recombinant cell vaccines containing the outer membrane protein K (*r-OmpK)* and the outer membrane protein J (*r-DnaJ)* to determine the protective efficacy against challenge with multiple *Vibrio* strains of *V. harveyi*, *V. alginolyticus*, and *V. parahaemolyticus.* We also investigated various immune responses induced by the vaccines, including the antibody production, immune related gene expression, and bactericidal effect, to gain insight into the responses that would elicit protection in the vaccinated fish. 

## 2. Materials and Methods

### 2.1. Fish Maintenance

Healthy *L. calcarifer* juvenile with overall mean weight and height with standard deviation of 10.41 ± 0.77 g, 5.38 ± 0.22 cm was purchased from a local breeder in Kota Bharu, Kelantan. Fish were acclimatized in two fiberglass tanks, each with size of 6.0 × 3.0 × 2.0 feet, and attached to a recirculating aquaculture system (RAS) at the Hatchery Unit of MARSLAB, Institute of Bioscience, UPM. At the same time, glass aquaria of 3.0 × 1.5 × 1.5 feet were filled with approximately 130 L of aerated seawater and were used to keep groups of fish during the experiment. The water temperature in the stocking tanks and glass aquaria was maintained between 26 and 30 °C, while the salinity was maintained between 28 and 30 ppm. The stocking density for each aquarium was 15 fish per tank and feeding was provided twice daily with commercial fish feed.

### 2.2. Preparation of Recombinant Cell as Formalin-Killed Vaccine

The gene encoding OMP-associated gene *OmpK* of *V. alginolyticus* VA2 and *DnaJ* of *V. harveyi* VH1 were constructed using pET32 Ek/LIC linearized vector (Novagen, Madison, WI, USA) and transformed into *E. coli* strain BL21 (DE3) (Data not shown). A novel protein band corresponding to 48.3 kda of *r-OmpK* (Accession no. KJ930426/AIG20833) and 49.0 kda of *r-DnaJ* (Accession no. KU144740/ANA53148) was detected in *E. coli* harboring PET32/EK LIC using SDS-PAGE and Western blotting (Data not shown). The cultures of the recombinant vaccine were harvested and killed in 0.5% formalin in phosphate buffered saline (PBS, pH 7.4; Sigma-Aldrich, St. Louis, MO, USA). Bacterial growth was observed to make sure all bacteria was killed prior injection.

### 2.3. Bacterial Strain and Culture Condition

The pathogenic Vibrio strains of wild type *V. harveyi*, *V. alginolyticus*, and *V. parahaemolyticus* strain were subculture into TCBS agar. The bacterial strain from TCBS agar plate was incubated at 30 °C with shaking at 150 rpm for 16 h. A single colony was identified as *V. harveyi* strain Vh1, V. alginolyticus strain VA2 and V. Parahaemolyticus strain VP were identified using PCR colony with gyrb gene. Then, the positive isolate of pathogenic Vibrio strains was sub-cultured and maintained in TSB + 1.5% NaCl (*w/v*) broth medium. The cells concentration was prepared to 10^8^ CFU/mL using the serial dilution protocols. In order to maintain the virulent properties of the pathogen used, the bacterial cultures were directly used during the pathogenic challenge test.

### 2.4. Experimental Design

A total of 750 *L. calcarifer* juveniles were used in this study. The fish were divided into five major groups and each group was further divided into triplicates of 50 fish. At the start of the experiment, Group 1 was exposed intraperitoneally to the recombinant cell vaccine containing *r-OmpK*, Group 2 to recombinant *r-DnaJ*, Group 3 to formalin-killed *V. harveyi,* Group 4 to BL21 cells, and Group 5 to PBS (Table 1). The spleen, kidney, and intestinal samples were collected from 3 fish of each group for qPCR gene quantification study at 0, 3, 6, 12, 24, 48, and 72 h post-vaccination. At weekly intervals, the serum, mucus, and gut lavage samples were collected from 3 fish of each group for antibody study.

On day 15 post-vaccination, 30 fishes from each group were challenged intraperitoneally with 10^8^ CFU/mL of live field-strain of *V. harveyi*, *V. alginolyticus*, and *V. parahaemolyticus* [11]. Clinical findings and mortality rate were recorded. All fish were killed on day 21 of the study and the gross lesions were recorded (Figure S1). The kidney, gut, and spleen of dead fishes were cultured on TCBS agar and incubated at 37 °C for 18 h to isolate bacteria. Suspected colonies of *Vibrio* were verified using colony PCR with gyrB primers [12]. Serum bactericidal activity of *L. calcarifer* juvenile was evaluated after post-vaccination and post challenge test. Water samples from the challenged tanks were collected at intervals of 24, 48, and 72 h post challenge for semi quantitative detection of bacterial in the seawater. All procedures in this study involving animals were approved by the Universiti Putra Malaysia (UPM) Institutional Animal Care and Use Committee, approval number: UPM/IACUC/AUP-R078/2019.

### 2.5. Serum Bactericidal Assay

The bactericidal assay was performed in sterile micro-tubes [13], in which 10 μL of 1 × 10^8^ CFU/mL of *V. harveyi* suspension and 10 μL of fish serum were incubated for 1 h at 37 °C. The resultant suspension was cultured in tryptone soya agar (TSA) and was incubated overnight at 37 °C, including a positive group that consisted of TSA plates containing bacteria and PBS suspension instead of the fish serum. Following overnight incubation, the CFU were manually counted. Results were expressed in values of CFU and serum bactericidal activity as the log_2_ value of percentage of colony forming units in the test group to that in the positive group: 1- CFU/positive (%) of seabass.

### 2.6. Agglutination Test

The serum, mucus, and gut lavage agglutinating titers were determined in a 96-well microtiter plate [14]. The assay was initiated by diluting samples at 1:1 ratio (50 mL of phosphate buffer: 50 mL of serum) into the first well. Two-fold serial serum dilutions were made by adding 50 mL of the diluted sample into the remaining wells containing 50 µL of PBS. Then, 50 µL of the inactivated bacteria at concentration of 1 × 10^8^ CFUs suspension was added into each well. The last well serves as a negative control, where there were only 50 mL PBS buffer. The micro plates were covered with plastic film and were incubated at room temperature for 16–18 h. The agglutination end point was established as the last serum dilution where agglutination was visible. Agglutination antibody titers were expressed as log2 (x + 1) of the reciprocal of the highest serum dilution showing visible agglutination as compared to the positive control [14]. 

### 2.7. Semi Quantitative PCR Detection of Bacteria from Water Samples

A method described by Jiang et al. [15] was used with some modification. Seawater samples were collected in a container in 100 mL quantities. The seawater was stored at −20 °C prior to DNA extraction. According to microbes preferred, which are the Vibrios, 10 mL of the seawater samples were mixed with 90 mL of specific culture media (TSB with 1.5 NaCl) and allowed to incubate in shaker at 37 °C for 16 h. Next, culture from the enriched seawater samples was washed with sterile distilled water at 4 °C, 10,000 rpm and bacterial DNA from the pelleted culture was extracted using the heat shock method for detection of Vibrios. The spleen of Vibrio-infected seabass was collected to serve as positive controls and the genomic DNA was extracted using Qiagen DNA extraction kit (Qiagen, Hilden, Germany). Genomic DNA from competent *E. coli* BL21 (DE3) cells was extracted using the heat shock method and used as negative controls. In order to detect the presences of Vibrios, the gyrB gene [16] was amplified from all the samples and the products were examined by 1% agarose gel electrophoresis containing ethidium bromide. The forward primer was 5′-GAGAACCCGACAGAAGCGAAG-3′ and the reverse primer was 5′-CCTAGTGCGGTGATCAGTGTTG-3′. The expected product size for Vibrio was approximately 400 bp.

### 2.8. Quantitative Detection of Recombinant Vaccines by Real Time qPCR

Total DNA was extracted from the spleen, kidney, and gut tissue samples using Qiagen DNA extraction kit (Qiagen, Hilden, Germany). Extracted DNA samples were checked for purity and concentration using spectrophotometry and stored in −20 °C. Quantitative PCR (qPCR) was conducted to measure the abundance of *r-OmpK* and *r-DnaJ* (Table 2) in the specific tissue samples. Each 10 µL reaction contained 1 µL of the target DNA extract (10 ng), 5 µL of IQ SYBR Green Supermix (Sigma-Aldrich, St. Louis, MO, USA), 1.25 µL of each primer (10 µm), and 1.5 µL of sterile distilled water. Thermal cycling conditions used were different for both the primers tested. Florescence (520 nm) was detected at the end of the elongation phase for each cycle. To evaluate amplification specificity, melting curve analysis was performed at the end of each PCR run. A melting curve profile was obtained by heating the mixture to 95 °C, cooling to 60 °C for each 10 s. To quantify unknown concentrations of each vaccine cells, a standard curve was generated by the amplification of a 10-fold dilution series of target vaccine in the presence of bacteria (control) DNA. The coefficient of correlation (R^2^) between the cycle threshold value (C_t_) and target DNA concentration was between 0.990–0.997, while the PCR efficiency was between 95–98%.

### 2.9. Total RNA Isolation, cDNA Synthesis, and RT-qPCR

Total RNA was prepared from kidney, intestine, and spleen at 0, 3, 6, 9, 12, 24, 48, and 72 h prime post-vaccination using TRIsure (Biorad, Hercules, CA, USA) lysates according to the manufacturer’s protocol. The cDNA was synthesized from total RNA samples with QuantiNova reverse transcription kit (Qiagen, St. Louis, MO, USA), which included random primers. The reaction was carried out in 10 μL reaction volumes using 36 columns Rotor Gene (Qiagen, Hilden, Germany) according to the manufactures protocol. The high-capacity cDNA reverse transcription kit could convert up to 1 μg of total RNA to cDNA and produced single-stranded cDNA, which was suitable for any quantitative PCR applications. The kit was stored at −20 °C and was allowed thawing on ice before use, except for the reverse transcriptase enzyme, which was kept at −20 °C at all times. The primers for each gene are given in Table 3. Each primer pair was tested with a set of cDNA and DNA samples to ensure that products could only be amplified from cDNA samples and not from genomic DNA under the conditions used. Primer efficiency was determined by serial dilutions of reference DNA and was used for quantification of the cDNA concentration. The expression of each gene was first normalized to that of ACTB and GAPDH, and expressed as copies number comparative to the expression level of non-vaccinated fish at the same time points. Subsequently, the gene copy number was calculated using the formula: number of copies = (DNA concentration × 6.022 × 10^23^)/(product length in bp × 1 × 10^9^ × 650) [18,19].

### 2.10. Statistical Analysis

The abundance of RT-qPCR gene expression level was subjected for one-way analysis of variance (ANOVA) and Tukey HSD post hoc test for comparison of mean and correlation analysis. Significant differences were considered at *p* < 0.05.

## 3. Results

### 3.1. Quantification of Targeted r-OmpK and r-DnaJ in Vaccinated Juvenile Seabass

**Figure 1:**
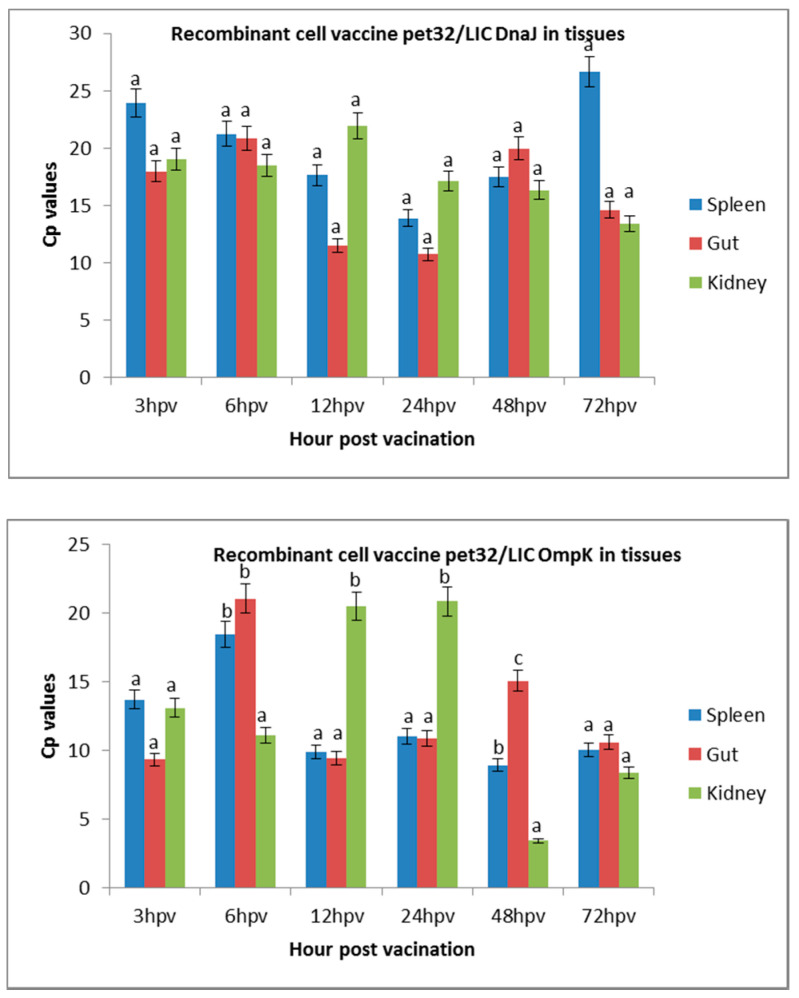
Expression of recombinant cell vaccines in the spleen, intestine, and kidney of vaccinated fish. The abundant expression in the spleen, gut, and kidney tissues was expressed as the crossing point (cp) value (the crossing point at which fluoresces crosses the threshold). The results are presented as means ± SD. A higher cp value indicates lower expression level. Significant differences are indicated by different letters (*p* < 0.05).

The presences of targeted *r-OmpK* and *r-DnaJ* were confirmed in the spleen, kidney, and intestine of juvenile seabass, and the crossing point (cp) values are presented in Figure 1. A higher cp value indicates a lower value of targeted vaccine. Therefore, the targeted level of *r-OmpK* in the kidney was significantly high at 3, 6, 24, and 72 h but was low at 12 and 24 h post-immunization. Although initially the spleen and gut showed low presences of *r-OmpK*, the targeted number increased after 6 h of vaccination and was significantly expressed in the kidney. On the other hand, the targeted *r-DnaJ* was observed to be less significant in all three tissues compared to the *r-OmpK* with cp values of above 15. Nevertheless, the presences of *r-DnaJ* were significantly increased in the gut and kidney at 12, 24, and 48 h post-vaccination. The least countenance was observed in the spleen tissue at 72 h. In the kidney, *r-DnaJ* was less expressed and the countenance was totally opposite to that of *r-OmpK*. Although the presences of *r-DnaJ* were significant in *r-DnaJ* vaccinated fish, there were no significant differences between the tissues. Overall, the uptakes of recombinant cell vaccines (*r-OmpK* and *r-DnaJ*) were different between different tissues in fish, which could influence the efficacy of the vaccine in fish.

### 3.2. Agglutinating Antibody Levels

**Figure 2:**
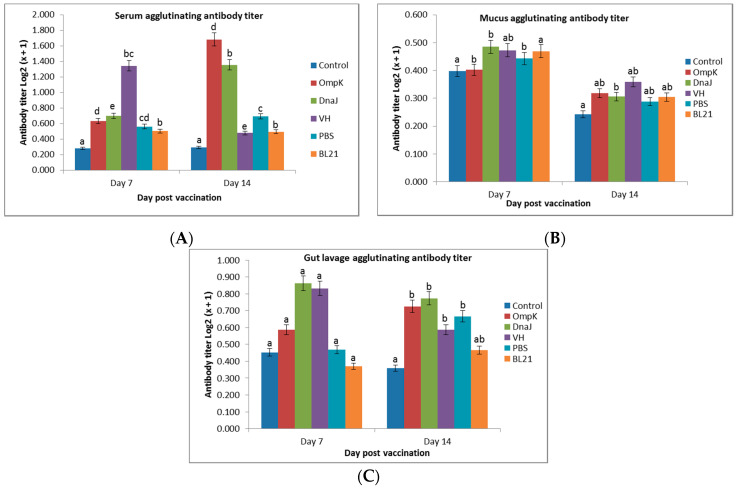
The agglutinating antibody titers in (**A**) serum, (**B**) mucus, and (**C**) gut lavage of immunized juvenile seabass at day 7 and 14 post-vaccination (mean ± sd). Significant differences are indicated by different letters (*p* > 0.05).

Increments of the antibody titer were observed in the serum and gut of vaccinated fish as early as day 7 and 14 post-vaccination. There were significantly (*p* < 0.05) high serum agglutination titers in both vaccinated groups (Figure 2A) compared to the control (healthy), PBS, and BL21 groups. However, there was a slight fluctuation of antibody level in the mucus after week 2 (Figure 2B). In general, there was a significant increase in serum antibody against *r-OmpK* (by 1.00) and *r-DnaJ* (0.7) after week 2 of immunization that peaked at day 7 and subsided by day 14. Similarly, the gut lavage fluid of *r-Ompk* and *r-DnaJ* vaccinated fish showed significant (*p* < 0.05) increase in the antibody titer by day 14 post-immunization (Figure 2C). Skin mucus, agglutinating antibody titer in vaccinated fish was low (Figure 2A–C) and antibody production was slightly declined at day 14 post-immunization. Although reduces after immunization at day 14, the agglutination antibody titer of vaccine *r-OmpK*, *r-DnaJ*, and *VH* were still higher than the control group. However, the average antibody levels were highest in *r-OmpK* and *VH* at day 14 post-vaccination.

### 3.3. Gene Expression Profilings

**Figure 3:**
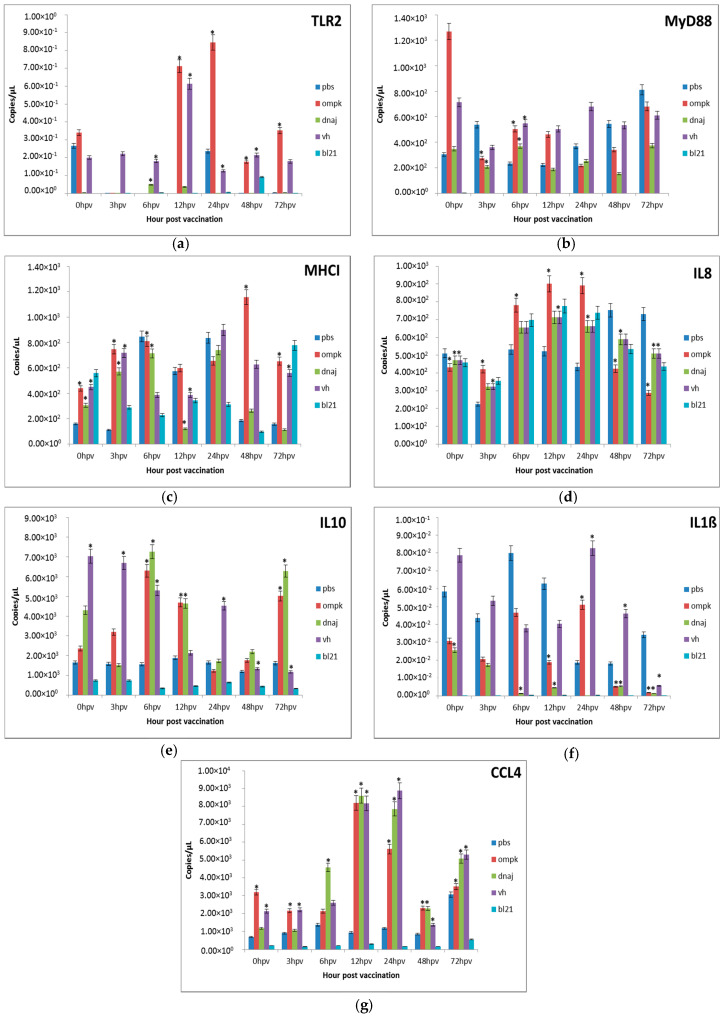
Comparative expression profiles of the antigen recognition-related genes (**a**) TLR2, (**b**) MyD88, (**c**) MHCI and pro-inflammatory cytokines (**d**) IL8, (**e**) IL10, (**f**) IL1ß, (**g**) CCL4 in the kidney of juvenile seabass after vaccination. Juvenile seabass were injected with 10^7^ CFU/mL of the inactivated pET-32/LIC-*OmpK* and pET-32/LIC-*DnaJ* recombinant cell, inactivated *E. coli* BL21 (DE3) and formalin-killed whole cell *V. harveyi* vaccines, while the unvaccinated control group was injected with PBS solution. Bars represent the mean copy number of three biological replicates and error bars represent standard deviation. Statistical significance was analyzed between the vaccinated and PBS control groups of seabass (* *p* < 0.05).

**Figure 4:**
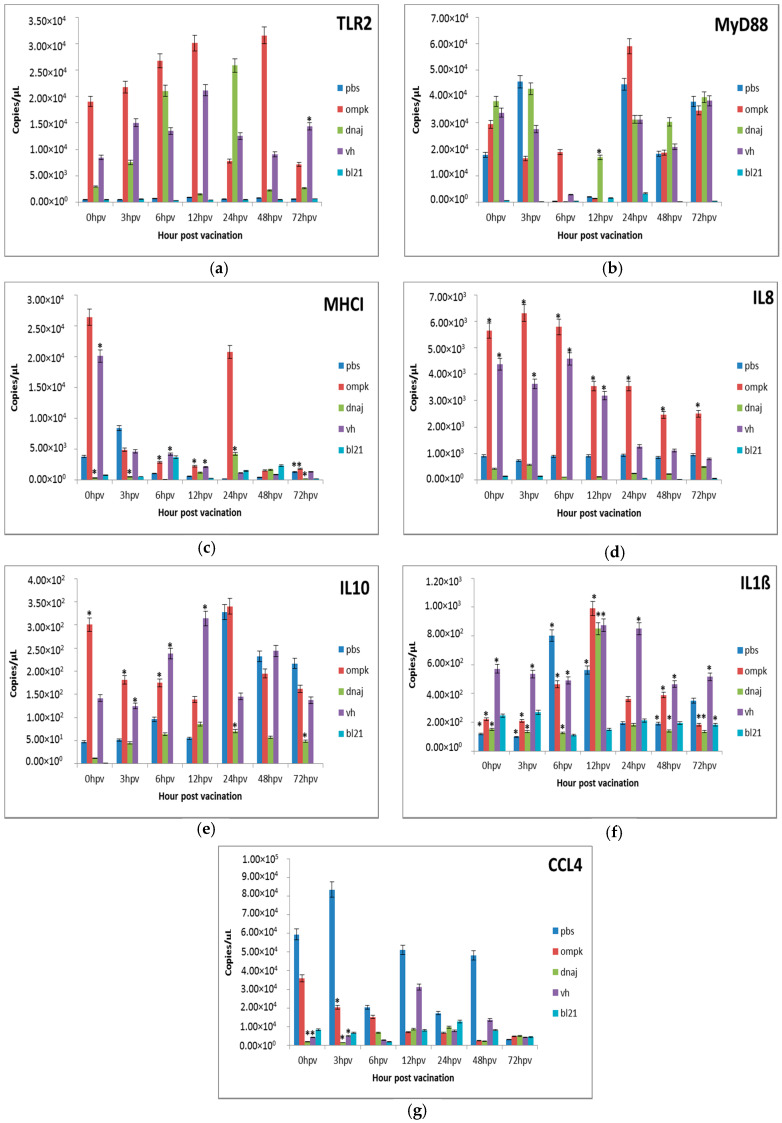
Comparative expression profiles of the antigen recognition-related genes (**a**) TLR2, (**b**) MyD88, (**c**) MHCI and pro-inflammatory cytokines (**d**) IL8, (**e**) IL10, (**f**) IL1ß, (**g**) CCL4 in the intestine of juvenile seabass after vaccination. Juvenile seabass were injected with 10^7^ CFU/mL of the inactivated pET-32/LIC-*OmpK* and pET-32/LIC-*DnaJ* recombinant cells, inactivated *E. coli* BL21 (DE3) and formalin killed whole cells *V. harveyi* vaccines, while unvaccinated fish were injected with PBS. Bars represent the mean copy numbers of three biological replicates and error bars represent standard deviation. Statistical significance was analyzed between the vaccinated and PBS vaccinated groups of seabass (* *p* < 0.05).

**Figure 5:**
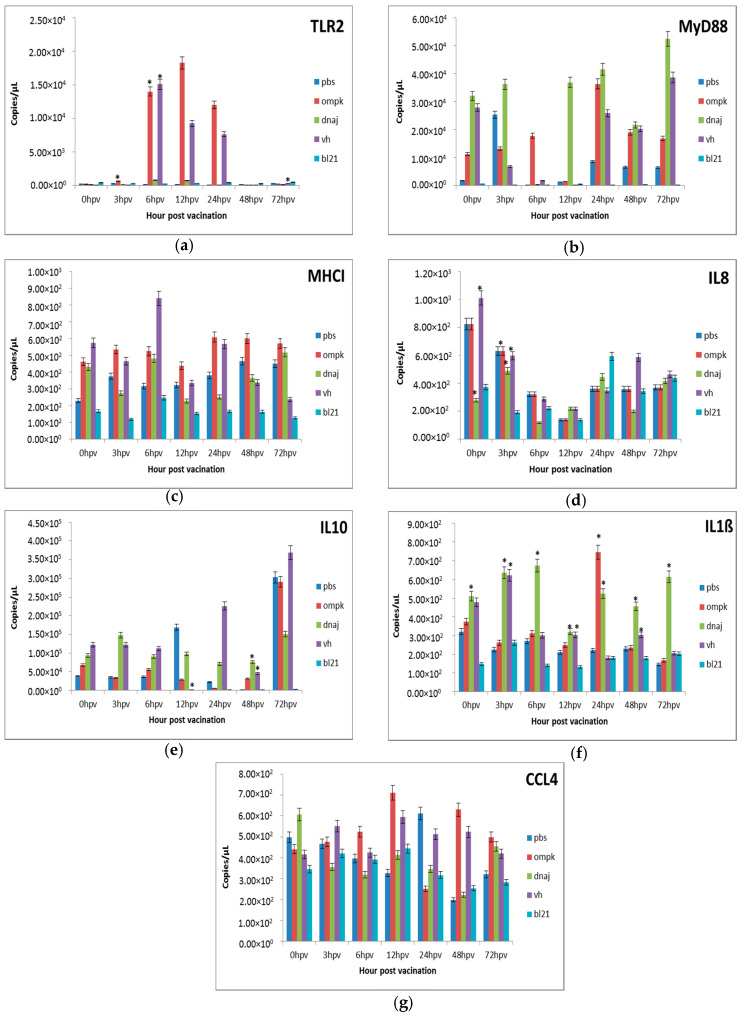
Comparative expression profiles of the antigen recognition-related genes (**a**) TLR2, (**b**) MyD88, (**c**) MHCI and pro-inflammatory cytokines (**d**) IL8, (**e**) IL10, (**f**) IL1ß, (**g**) CCL4 in the spleen of juvenile seabass after vaccination. Juvenile seabass were injected with 10^7^ CFU/mL of the inactivated pET-32/LIC-*OmpK* and pET-32/LIC-*DnaJ* recombinant cells, inactivated *E. coli* BL21 (DE3) and formalin killed whole cells *V. harveyi* vaccines, while the unvaccinated control group was injected with PBS solution. Bars represent the mean copy numbers of three biological replicates and error bars represent standard deviation. Statistical significance was analyzed between the vaccinated and PBS vaccinated groups of seabass (* *p* < 0.05).

To investigate whether *r-Ompk* and *r-DnaJ* were recognized by the host, we measured the expression of the genes in the kidney, spleen, and gut tissues of juvenile bass at 0, 3, 6, 12, 24, 48, and 72 h post-vaccination (Figure 3, Figure 4 and Figure 5).

#### 3.3.1. Gene Expression in the Kidney

In this study, there were significant (*p* < 0.05) expressions of the toll-like receptor 2 (TLR-2) from 12 to 72 h post-vaccination with an increase of *r-OmpK* in the kidney between 12 and 24 h post-vaccination (Figure 3a). Vaccination with killed *V. harveyi* resulted in similar (*p* > 0.05) expression as vaccinated groups. On the other hand, the expression of the intracellular TLR adaptor molecule, the MyD88, was higher (*p* < 0.05) following vaccination with *r-OmpK* and dramatic reduction could be observed thereafter (3 h onwards) in the kidney. During the initial 3 h, the MyD88 expression was significantly (*p* < 0.05) high in kidney immunized with *r-OmpK*, *r-DnaJ*, and *V. harveyi* vaccines (Figure 3b). The expression of MHCII was significantly (*p* < 0.05) enhanced in the kidney immunized with *r-OmpK*, *r-DnaJ*, and *V. harveyi* in the first 6 h, but the expression was significantly (*p* < 0.05) higher in *r-OmpK* and peaked at 48 h (Figure 3c). The IL-8 was expressed at high level in the kidney of the PBS group and showed significant (*p* < 0.05) up-regulation for the *r-OmpK* group at 6 to 24 h (Figure 3d). The peak IL10 expression for *r-OmpK* and *r-DnaJ* vaccines was at 6 and 12 h, respectively, followed by gradual drop in expression thereafter (Figure 3e). Even though IL10 produced significant (*p* < 0.05) expression in the kidney, vaccination with *V. harveyi* showed decreasing expression with time. The minimal expression of the IL-1ß gene was also observed in the *V. harveyi*-vaccinated kidney, while significant (*p* < 0.05) expression was detected at 48 and 72 h for *r-OmpK* and *r-DnaJ*, respectively (Figure 3f). Significant (*p* < 0.05) expression was also observed for CCL4 in the kidney following vaccination (Figure 3g). The expression increased after vaccination with *r-OmpK*, *r-DnaJ*, and *V. harveyi*, with expressions of five-times higher at 12 and 24 h post-vaccination.

#### 3.3.2. Gene Expression in the Intestine

The TLR and MyD88 genes were constitutively expressed in the intestine following vaccination (Figure 4a,b). Expression of TLR2 was increased at 0.5, 1.0, and 1.5 × 10^4^ copies/µL at 3, 6, and 12 h post-vaccination with *r-OmpK*. Interestingly, TLR2 in the intestine showed fast up-regulation at 48 h with drastic reductions at 24 and 72 h. In addition, a significant (*p* < 0.05) expression was observed only at 72 h following vaccination with *V. harveyi*. Meanwhile, vaccination with *r-DnaJ* resulted in a slight up-regulation within the first three time points, significantly (*p* < 0.05) less expressed compared to *r-OmpK* (Figure 4a). On the other hand, MyD88 showed massive (*p* < 0.05) down regulations at 6 and 12 h, but increased back at 24 and 72 h (Figure 4b). The MHCII was constitutively expressed in the intestine at varying degrees during the experimental period (Figure 4c). The expression patterns of IL8 revealed significant (*p* < 0.05) in the intestine vaccinated with *r-OmpK* and *V. harveyi* (Figure 4d), but the expression was reducing after 12 h. Following vaccination with *r-DnaJ*, the IL8 expressions were extremely (*p* < 0.05) low and most similar (*p* > 0.05) to the PBS-vaccinated fish. Meanwhile, the expression of IL10, an anti-inflammatory cytokine that is involved in the pro-inflammatory outcome control, was significant (*p* < 0.05) between 0 and 12 h following vaccination with *r-OmpK* and *V*. *harveyi* (Figure 4e) and reached peak at 24 h. Vaccination with *r-DnaJ* resulted in constant expression throughout the experimental period. The expression of IL-1ß was significantly (*p* < 0.05) enhanced following vaccination with *r-OmpK* and *V. harveyi* up to 12 h, subsequently declined until 72 h post-vaccination (Figure 4f). Consequently, CCL4 was weakly expressed after vaccination, instead high regulations were attained following exposure to PBS (Figure 4g). Low but significant (*p* < 0.05) expression was observed immediately after vaccination with *r-Ompk*, *r-DnaJ*, and *V. harveyi*.

#### 3.3.3. Gene Expression in the Spleen

The TLR2 gene was weakly expressed in the spleen and increasing expressions were observed only in fish vaccinated with *r-Ompk* and *V. harveyi* between 6 and 24 h (Figure 5a). On the other hand, the MyD88 (Figure 5b) was generally expressed in all vaccinated groups of *r-OmpK*, *V.harveyi*, and *r-DnaJ*, but with weak expressions at 6 and 12 h. The MHCI was constitutively expressed in the spleen and the level of expression was maintained until 72 h (Figure 5c). Even though, the expression of IL8 was significantly (*p* < 0.05) enhanced initially, there was slight reductions at 6 and 12 h similar (*p* > 0.05) to the PBS group (Figure 5d). The IL10 was highly (*p* < 0.05) expressed in the spleen, initially low level up to 6 h but showed massive up-regulation at 72 h (Figure 5e). In contrast, CCL4 was mostly up-regulated (*p* < 0.05) throughout the experimental period (Figure 5g). By 6 h, more changes in gene expression were apparent in the *r-OmpK* and *V. harveyi* vaccinated fish. On the other hand, the pro-inflammatory cytokine IL-1ß showed a significant up-regulation following vaccination with with *r-DnaJ* (Figure 5f). A peak in expression for IL-1ß was observed at 24 h for the *r-OmpK* group.

### 3.4. Clinical Symptoms of Infected Fish

**Figure 6:**
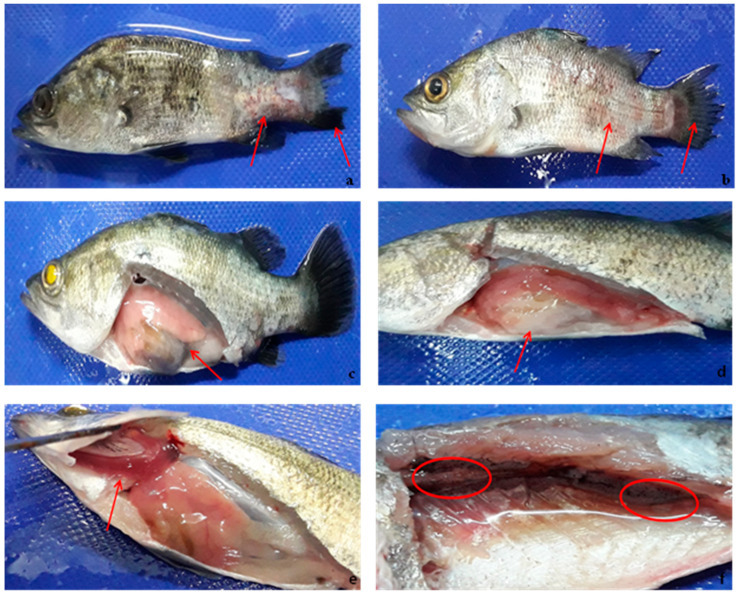
Juvenile seabass experimentally challenged with pathogenic V. harveyi showing (**a**,**b**) hemorrhagic skin, eye opacity, tail and fin rot, (**c**) Infected and swollen gonad, (**d**) Swollen intestine, (**e**) Pale and discolored of gill, and (**f**) Black spots.

Following intraperitoneal challenge with 10^8^ cfu/mL of live *V. harveyi*, *V. parahaemolyticus*, and *V. alginolyticus*, typical symptoms of vibriosis were observed as early as 24 h in the juvenile seabass exposed to PBS. No obvious sign was observed in the vaccinated juvenile seabass (the *r-OmpK*, *r-DnaJ*, and *V. harveyi* groups). The unvaccinated juvenile bass showed different degrees of signs within the first 24 h post-infection with *V. harveyi*. They exhibited sluggish movement, reduces mucus secretion, peeling of scales, eye opacity, hemorrhagic skin, particularly at the base of fins and tail, and fin rot (Figure 6a,b). Necropsy revealed pale gill (Figure 6e), black spots (Figure 6f), and dilated intestine and swollen gonads (Figure 6c,d). Furthermore, behavioral changes included inappetence and reduced feeding, and abnormal swimming patterns. Following infection with *V. alginolyticus* and *V. parahaemolyticus*, the unvaccinated juvenile seabass did not show any sign for the first 24 h post-challenge.

### 3.5. Relative Percentage Mortality

In this experiment, mortalities were observed in the non-vaccinated juvenile seabass that were challenged with *V. harveyi*. The cumulative rate of mortality reached 100% at 48 and 36 h in PBS and BL21 groups, respectively. Those challenged with *V. alginolyticus* and *V. parahaemolyticus* started to die at 24 h and reached peak at 87% and 80% of accumulative mortality at 72 h, respectively. No mortality was observed in the groups vaccinated with *r-OmpK* and *V. harveyi* and challenged with *V. alginolyticus* and *V. parahaemolyticus*. However, the *r-DnaJ* vaccinated group showed cumulative rate of mortality from 33% at 24 h to 67% at 72 h post infection (Table 4). Therefore, fish vaccinated with *r-OmpK* and *V. harveyi* and challenged with *V. harveyi* have yielded the RPS value of 87% and 93%, respectively, whereas, the *r-DnaJ* resulted in 33% RPS value at 72 h post challenge with *V. harveyi* and 23% and 17% following challenge with *V. alginolyticus* and *V. parahaemolyticus*, respectively.

### 3.6. Bacterial Isolation

**Figure 7:**
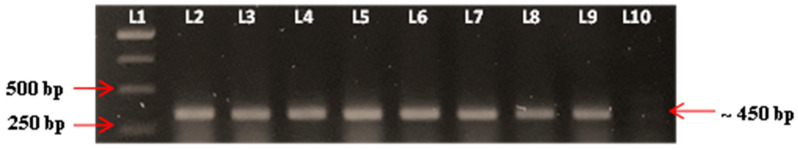
Confirmations of *Vibrio* spp. isolated from dead experimental fish by using PCR, gyrB forward and reverse primers. Lane 1: 1 kb DNA ladder marker (Fermentas, Waltham, MA, USA), Lane 2: Positive control (ATCC of *V. harveyi*), Lane 3–9: Positive colonies of vibrio spp., Lane 10: Negative control.

Suspected *Vibrio* species were successfully isolated from the spleen, kidney, and intestine of challenged fish (Table 5). Bacterial growths were readily observed in fish infected with *V. harveyi*, and vaccinated fish showed less bacterial growth compared to the control PBS group. Massive bacterial growth was observed in the kidney and spleen, while few were isolated from the intestine of control fish, while reduced numbers of bacteria were isolated from the vaccinated fish (Figure S2). The *GyrB* gene produced positive amplicons at 450 bp as shown in Figure 7.

### 3.7. Serum Bactericidal Activity

**Figure 8:**
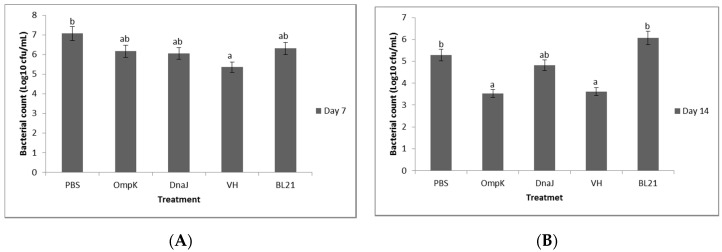
Bacterial count (cfu/mL) in the serum of non-vaccinated and vaccinated juvenile seabass at (**A**) days 7 and (**B**) day 14 post-vaccination. Significant differences are indicated by different letters (*p* < 0.05).

**Figure 9:**
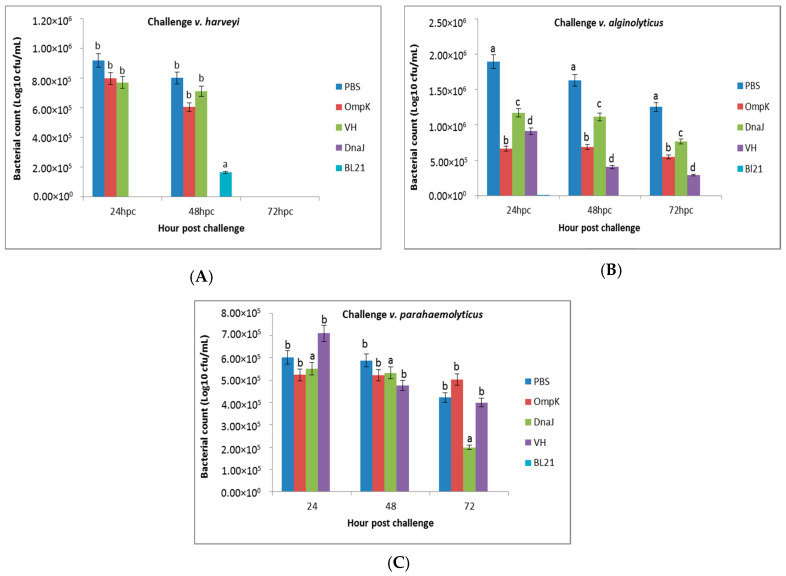
Bacterial count (cfu/mL) in serum of non-vaccinated and vaccinated juvenile seabass at 24, 48, and 72 h post-challenge with (**A**) *V. harveyi*, (**B**) *V. parahaemolyticus*, and (**C**) *V. alginolyticus*. Significant differences are indicated by different letters (*p* < 0.05).

The serum samples from vaccinated fish and those that survived the experimental challenge were subjected to bactericidal activity. The bacterial growth was reduced when subjected to the post-vaccination sera of days 7 and 14, and to post-challenge sera of 24, 48, and 72 h (Figure 8 and Figure 9). The reduction was much higher with the serum collected from fish that were vaccinated with *r-OmpK* and *V. harveyi* after 14 days of exposure (Figure 8). On the other hand, the growth of *V. harveyi* was inhibited by the serum of fish vaccinated with *r-OmpK* and *V. harveyi* at 24 and 48 h post-challenge (Figure 9A). The bacterial count using sera challenged with *V. parahaemolyticus* and *V. alginolyticus* resulted in a stepwise decrease in bacterial survival for group vaccinated with *r-OmpK* and *V. harveyi*, respectively (Figure 9B,C).

### 3.8. Pathogen Shedding

Semi-quantitative PCR detection of *Vibrio* from the water of each group is summarized in Table 6. Faint band representing *Vibrio* spp. could be detected in the water of vaccinated and challenged groups at 24 h post-challenged, while a much brighter band was observed in the water of the control unvaccinated and challenged group at the same time point. At 72 h, the band intensity increased for all groups, with the control unvaccinated and challenge group showing much stronger band.

## 4. Discussion

Vibriosis is the most common bacterial disease affecting mariculture fishes worldwide. *Vibrio harveyi*, *V. alginolyticus*, and *V. parahaemolyticus* are the dominant *Vibrio* species that cause massive mortalities in cultured seabass and other marine fish [23]. In this study, juvenile seabass were vaccinated with the *r-OmpK*, *r-DnaJ*, and *V. harveyi* vaccines, and the challenge trial revealed that fish vaccinated with *r-DnaJ* showed a higher rate of mortality from 25% at 24 h to 70% at 72 h post-challenge with symptoms typical of vibriosis developed after the challenge with *V. harveyi* [22,24,25]. The other two vaccines resulted in no mortality except those that were challenged with *V. harveyi*, which showed <13% mortality. In this study, all the infected juvenile seabass showed typical clinical signs and post-mortem lesions as reported in naturally infected fish.

Immunization with recombinant cell vaccine containing *r-OmpK* gene in this study has been effective in inducing immune responses that eventually protect the fish against bacterial challenge. High antibody titers were observed in serum and gut of vaccinated groups as early as day 7 post-vaccination. Similarly, an experiment involving *Seriola quinqueradiata* vaccination against *Photobacterium damsela piscicida*, detected high antibody levels three and four weeks after immunization [26] and tilapia vaccinated with 1 × 10^8^ CFU bacteria/mL of *A. hydrophila* showed high antibody titer after 14 days [27]. Findings in this study suggest that the agglutinating antibody played a major role in host defense, particularly during the initial phase of the infection [28,29,30,31]. However, interpreting the mucus antibody activity is a challenge since the quantity and concentration of antibodies in the mucus sample is limited, existent of other proteins in the mucus may interrupt the results, and also antibodies produced by mucosal immune response exist as different isotypes from the serum antibodies.

To date, the mechanisms of disease resistance in vaccinated fish remain largely unknown. Therefore, understanding genes and gene expression are crucial. This study revealed that spleen was a crucial organ for the fish to generate antibody [32], while the kidney and intestine were among the essential immune organs that were involved in fish resistance against invading pathogen. The TLR2 was highly expressed in the intestine, followed by the spleen and kidney following vaccination with *r-OmpK* and *V. harveyi* as observed in zebrafish [33], fugu [34], rainbow trout [35], channel catfish [36], common carp [37], rohu [38], grass carp [39], orange-spotted grouper [40], large yellow croaker [41], miiuy croaker [42,43], Japanese flounder [44], and gibel carp [45]. However, TLRs are important for recognition of a series of Gram-positive pathogens, and since *Vibrio* is a Gram-negative bacterium, lower expression pattern of TLR2 was expected at the initial phase post-vaccination.

The major histocompatibility complex (MHC) molecules play a central role in adaptive immunity. They are immunoglobulin super family member proteins that interact with T-cells through a specific T-cell receptor (TCR) in order to initiate immune responses [46] and both types of MH receptors (MHC I and II) are present in teleosts [47]. Following vaccination in this study, the expression of MHCI was clearly expressed in the kidney and spleen, while a minimal expression was observed in intestine tissues, indicating a powerful initiation of cellular immune response upon recognition of the recombinant cell vaccines at the early stage of vaccination. The transcription levels of this gene in the vaccinated group showed a specific cellular immunity that prevented the pathogen from invading the juvenile bass and damaging the host tissue.

One of the significant outputs of antigenic stimulation is the production and secretion of cytokines and chemokines by macrophages and dendritic cells that help to prompt and uphold inflammation. Thus, the expressions of several cytokines and chemokines involved in pro-inflammatory responses were determined in this study. The mRNA levels of IL-8, IL-10, IL-1ß, and CCL4 were significantly expressed in the intestine, spleen, and kidney following vaccination with *r-OmpK* and *V. haveyi*. IL-1ß, which was highly expressed in vaccinated fish, is a typical pro-inflammatory cytokine produced by several cells like macrophages [35], which allows the growth and proliferation of immune cells [36] and it is believed to act as an important signal for the early immune response. Up-regulation of IL8 following vaccination with *r-OpmK* facilitates migration of neutrophil in pathogen resistance [48]. Similar phenomenon was observed in rainbow trout vaccinated with *Yersinia ruckeri* bacterin [49]. Basically, analyses of gene expressions in the spleen, kidney, and intestine of juvenile bass post-vaccination demonstrate that the three organs played a major role in the main portal entry for the recombinant cell vaccines, particularly the *r-OmpK*. Amidst the uptakes of recombinant cell vaccines (*r-OmpK* and *r-DnaJ*) by different tissues, could also influence the expression of immune related genes in the vaccinated fish.

Besides the explication of the functional genomic aspects of juvenile seabass immune response, this study also revealed the semi-quantitative amount of the pathogen within the holding seawater. Diseased fish are expected to shed the pathogen into the water. Several authors have successfully detected the pathogen from the environmental samples using the PCR-dependent method [50]. We found that the *Vibrio* species (*V. harveyi*, *V. parahaemolyticus*, and *V. alginolyticus*) could be detected in the holding water of challenged groups, but less bacteria were detected in the water of the vaccinated group. This might indicate the less shedding of *Vibrio* into the water by the vaccinated fish. Therefore, vaccination not only stimulated an effective immune response against the pathogen, but also decreased the horizontal spread of the pathogen by reducing the bacterial shedding.

## 5. Conclusions

In this study, the recombinant cells vaccine containing *r-OmpK* worked efficiently against all three *Vibrio* strains. Vaccinating juvenile seabass induces the expression of immune-related genes in the spleen, liver, and intestine, indicating that the organs play a major role in fish immune response. The high antibody titer contributed to the higher post-challenge survival rate of the fish.

## Figures and Tables

**Table 1:** Experimental group for fish vaccination study.

Treatment Groups	Vaccines	Dosages
Group 1	Recombinant cell pet32/LIC *OmpK* vaccine (*r-OmpK*)	10^7^ cfu/mL
Group 2	Recombinant cell pet32/LIC *DnaJ* vaccine (*r-DnaJ*)	10^7^ cfu/mL
Group 3	Whole cells-killed *V. harveyi* vaccine (VH), positive control	10^7^ cfu/mL
Group 4	BL21 (DE2) vaccine (BL21), negative control	10^7^ cfu/mL
Group 5	Phosphate buffered saline (PBS), control	0.01 M

**Table 2:** Recombinant cell vaccine sequences for *r-OmpK* and *r-DnaJ*.

No.	Vaccines	Forward Primer (5′–3′)	Reverse Primer (5′–3′)	Reference
1	*r-OmpK*	GACGACGACAAGATGCGTAAATCACT	GAGGAGAAGCCCGGTTAGAACTTGTA	Qian et al. [17]
2	*r-DnaJ*	GACCACGACAAGATGCATATTTTTGGC	GAGGAGAAGCCCGGTTATTTAAAGCC	

**Table 3:** List of genes for quantification of immune related genes in sea bass against vibriosis.

No.	Genes	Forward Primer (5′–3′)	Reverse Primer (5′–3′)	Reference
1	ACTB *	taccaccggtatcgtcatgga	Ccacgctctgtcaggatcttc	Paria et al. [20]
2	GAPDH *	cgcttcctgcacaaccaact	Gtggcagtgatggcatgaac	
3	TLR-2	Tctccgtcttggtttcac	Ggtcccacagttgagtatg	Dahai Yang et al. [21]
4	MHC I	ggctgtttttgccgctct g	Gtggacaggtctggataaag	
5	Myd88	Aacaacttcgctggataa	Gttactggaatcgcctca	
6	IL-8	Cttccctccaagcccacag	Gatccgggcattcatgg	Reyes-López et al. [22]
7	IL-1B	Atctggaggtggtggacaaa	Agggtgctgatgttcaaacc	
8	IL-10	Cgaccagctcaagagtgatg	Agaggctgcatggtttctgt	
9	CCL4	Tcctcgtctcactctgtctgt	Gacctgccactgtcttcagc	

* Housekeeping gene.

**Table 4:** Relative percentage survival (RPS) of vaccinated *Lates calcarifer* at 72 h post infection with multiple pathogenic *Vibrio* strain.

Challenged Bacteria	*V. harveyi* (10^8^ CFU/mL)			*V. alginolyticus* (10^8^ CFU/mL)			*V. parahaemolyticus* (10^8^ CFU/mL)		
Vaccination Treatment	Survival (%)	Mortality (%)	RPS (%)	Survival (%)	Mortality (%)	RPS (%)	Survival (%)	Mortality (%)	RPS (%)
*r-OmpK*	87	13	87	100	0	100	100	0	100
*r-DnaJ*	33	67	33	33	63	23	33	67	17
VH	93	7	93	100	0	100	100	0	100
*E. coli* BL21(DE3)	0	100	0	0	100	0	0	100	0
PBS	0	100	n.a	13	87	n.a	20	80	n.a

Notes: n.a = not applicable, Number of vaccinated fish = 50, Number of challenged fish = 30.

**Table 5:** Detection of yellow colonies of *Vibrio* on TCBS agar, isolated from the spleen, intestine, and kidney of fish challenged with *V. harveyi*, *V. alginolyticus*, and *V. parahaemolyticus*.

Treatments	*Vibrio harveyi* (10^8^ cfu/mL)			*Vibrio alginolyticus* (10^8^ cfu/mL)			*Vibrio parahaemolyticus* (10^8^ cfu/mL)		
	Spleen	Kidney	Intestine	Spleen	Kidney	Intestine	Spleen	Kidney	Intestine
PBS	+++	+++	+++	+++	+++	++	+++	+	-
*r-OmpK*	+++	+++	-	++	-	-	+	-	-
*r-DnaJ*	+++	+++	-	++	++	+	+	-	-
VH	++	-	-	+	-	-	+	-	-
*E.coli* BL21 (DE3)	+++	+++	+++	+++	+++	++	++	+	+

Note: Symbols showing the condition of growth for each tissue on TCBS plate. +++ Massive growth, ++ Moderate growth, + Minimum growth, - No growth.

**Table 6:** Semi-quantitative PCR detected variable amount of *V. harveyi*, *V. parahaemolyticus*, and *V. alginolyticus* from the seawater samples of challenged fish.

Treatments	*V. harveyi*			*V. alginolyticus*			*V. parahaemolyticus*		
	24hpc	48hpc	72hpc	24hpc	48hpc	72hpc	24hpc	48hpc	72hpc
+ve control *	+++	+++	+++	+++	+++	+++	+++	+++	+++
PBS	++	++	+++	+++	+++	+++	+	+++	+++
*r-OmpK*	+	++	++	++	++	++	-	-	++
*r-DnaJ*	+	++	+++	++	+++	+++	-	+++	+++
VH	+	-	++	+	++	++	-	-	-
-ve control *	-	-	-	-	-	-	-	-	-

Note: +++ Strongly amplified band, ++ Moderately amplified band, + Faint amplified band, - No amplification, * +ve control: Genomic DNA extracted from spleen tissue, * -ve control: Genomic DNA of *E.coli* BL21 (DE3).

## Supplementary Materials

The following are available online at https://www.mdpi.com/2076-393X/8/4/660/s1, Figure S1: The timeline study of in vivo pathogenicity and safety level in *Lates calcarifer*, Figure S2: Growth of yellow colonies on thiosulfate-citrate-bile salts-sucrose (TCBS) agar from culture of isolates from spleen, intestine and kidney of challenged fish of (A) *V. harveyi,* (B) *V. alginolyticus* and (C) *V. parahaemolyticus*.
