# Supplementary material for: Elucidating the Efficacy of Vaccination against Vibriosis in Lates calcarifer Using Two Recombinant Protein Vaccines Containing the Outer Membrane Protein K (r-OmpK) of Vibrio alginolyticus and the DNA Chaperone J (r-DnaJ) of Vibrio harveyi
Source: Vaccines (Basel). 2020 Nov 6;8(4):660. doi: 10.3390/vaccines8040660 (PMC7711666; doi:10.3390/vaccines8040660)
Supplement: Supplementary file 1 [file vaccines-08-00660-s001.pdf]

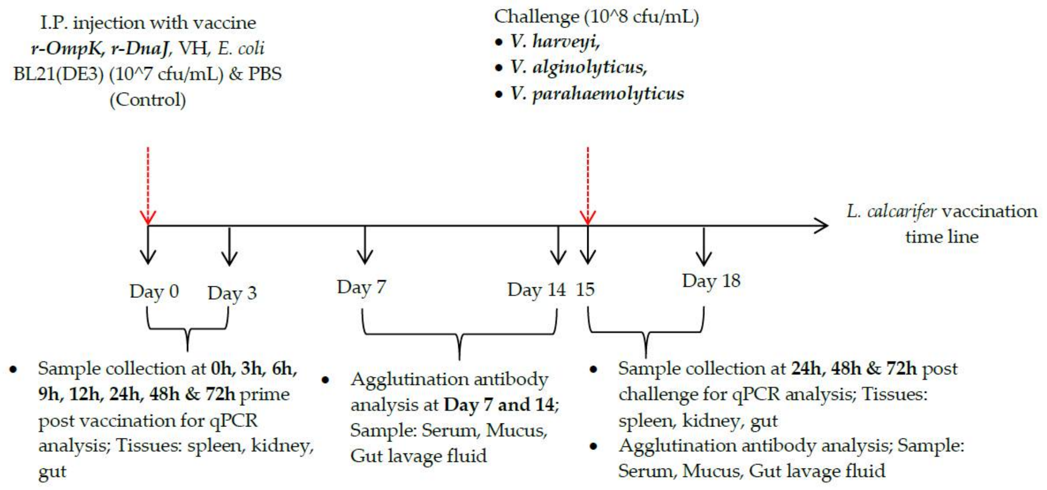


**Figure S1.** The timeline study of in vivo pathogenicity and safety level in *Lates calcarifer*.

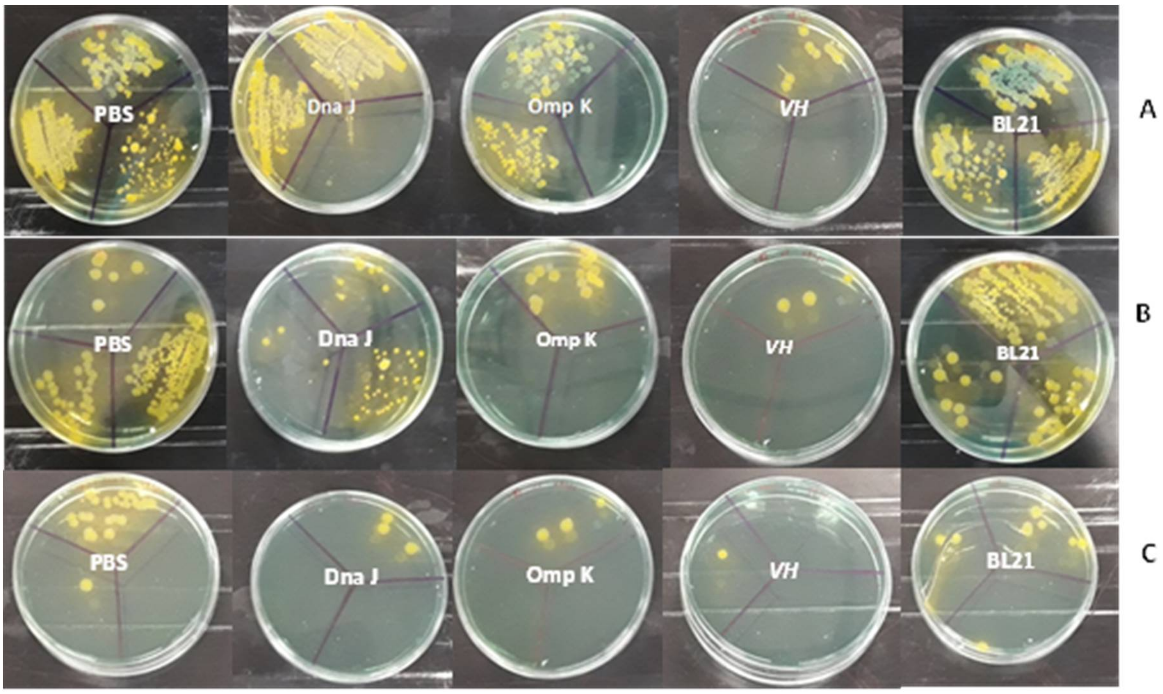


**Figure S2.** Growth of yellow colonies on thiosulfate-citrate-bile salts-sucrose (TCBS) agar from culture of isolates from spleen, intestine and kidney of challenged fish of (**A**) *V. harveyi,* (**B**) *V. alginolyticus* and (**C**) *V. parahaemolyticus*.
